# The Protective Effect of Human Umbilical Cord Blood CD34+ Cells and Estradiol against Focal Cerebral Ischemia in Female Ovariectomized Rat: Cerebral MR Imaging and Immunohistochemical Study

**DOI:** 10.1371/journal.pone.0147133

**Published:** 2016-01-13

**Authors:** Ching-Chung Liang, Ho-Ling Liu, Shuenn-Dhy Chang, Sheng-Hsien Chen, Tsong-Hai Lee

**Affiliations:** 1 Female Urology Section, Department of Obstetrics and Gynecology, Chang Gung Memorial Hospital Linkou Medical Center, Taoyuan, Taiwan; 2 College of Medicine, Chang Gung University, Taoyuan, Taiwan; 3 Department of Imaging Physics, The University of Texas M.D. Anderson Cancer Center, Unit 1472, 1515 Holcombe Boulevard, Houston, TX, 77030, United States of America; 4 Institute of Biotechnology, Southern Taiwan University of Science and Technology, Tainan, Taiwan; 5 Stroke Section, Department of Neurology and Stroke Center, Chang Gung Memorial Hospital Linkou Medical Center, Taoyuan, Taiwan; National University of Singapore, SINGAPORE

## Abstract

Human umbilical cord blood derived CD34^+^ stem cells are reported to mediate therapeutic effects in stroke animal models. Estrogen was known to protect against ischemic injury. The present study wished to investigate whether the protective effect of CD34^+^ cells against ischemic injury can be reinforced with complemental estradiol treatment in female ovariectomized rat and its possible mechanism. Experiment 1 was to determine the best optimal timing of CD34^+^ cell treatment for the neuroprotective effect after 60-min middle cerebral artery occlusion (MCAO). Experiment 2 was to evaluate the adjuvant effect of 17β-estradiol on CD34^+^ cell neuroprotection after MCAO. Experiment 1 showed intravenous infusion with CD34^+^ cells before MCAO (pre-treatment) caused less infarction size than those infused after MCAO (post-treatment) on 7T magnetic resonance T2-weighted images. Experiment 2 revealed infarction size was most significantly reduced after CD34^+^ + estradiol pre-treatment. When compared with no treatment group, CD34^+^ + estradiol pre-treatment showed significantly less ADC reduction at 2 h and 2 d, less CBF reduction at 2 h and less hyperperfusion at 2 d. The immunoreactivity of c-Fos, c-Jun and GFAP was attenuated, and BDNF showed significant recovery from 2 h to 2 d after MCAO, especially after CD34^+^ + estradiol pre-treatment. The present study suggests pre-treatment with CD34^+^ cells with complemental estradiol can be most protective against ischemic injury, which may act through stabilization of cerebral hemodynamics and normalization of the expressions of immediate early genes and BDNF.

## Introduction

Stroke is ranked as one of the leading causes of death, and the poststroke neurological disability is the most important problem to cause handicap worldwide. Until now, there is limited preventive treatment which could be effective against neuronal damage. Human umbilical cord blood (HUCB) derived CD34^+^ stem cells can exhibit neuronal or glial cell properties under defined culture conditions [1] and have been reported to mediate therapeutic effects in animal model of stroke [2,3]. Early treatment with HUCB cells may facilitate functional recovery after middle cerebral artery occlusion (MCAO) in rats [4], and the infarction volume measured by histological sections revealed an inverse relationship with HUCB cell dose [5].

Glial fibrillary acidic protein (GFAP) is a marker for astrocyte, and reactive astrocytosis can occur after ischemic injury [6]. Brain-derived neurotrophic factor (BDNF) is known to be protective against neuronal damage in in vivo and in vitro studies [7,8]. Combined intravenous treatment with HUCB cells and mannitol was found to significantly increase cerebral BDNF, which correlated positively with reduced cerebral infarction and improved behavioral functions in a MCAO rat model [9]. Immediate early genes, c-Fos and c-Jun, can be up-regulated by various forms of brain injury, and is involved in programmed cell death. In women after menopause, the production of ovarian hormones, progesterone and estrogen, decreases and the risk for cerebral ischemia increases. Estrogen has been known to confer natural protection to premenopausal women from ischemic stroke. Estrogen administered before or after ischemia has demonstrated protective effect against ischemic damage not only in normal rats [10,11] but also in ovariectomized rats [12,13], and is found to selectively attenuate the injury-induced increase of c-Fos after MCAO [14].

In recent decades, magnetic resonance (MR) imaging has obtained considerable interest in the evaluation of cerebral hemodynamic changes after ischemic injury [15,16]. There is no study using MR diffusion and perfusion to study the potential mechanism of HUCB derived CD34^+^ cells and/or estrogen treatment on cerebral hemodynamics and correlate with the expression of immediate early genes, BDNF and GFAP after ischemic injury. Previous reports usually used normal rat to study the protective effect of HUCB derived CD34^+^ cell against ischemic injury. The present study wished to test the hypothesis whether in ovariectomized rats, complemental estradiol can enhance the neuroprotection of CD34^+^ cell, and whether this neuroprotection may involve the stabilization of cerebral hemodynamics and normalization of the expression of immediate early genes and BDNF.

## Materials and Methods

### Animal Model

All protocols were approved by Institutional Ethics Committee for the Care and Use of Experimental Animals and Institutional Review Board of Chang Gung Memorial Hospital. Experimental animals from the National Laboratory Animal Center were maintained at 21–23°C room temperature and 47% humidity with 12-h light-dark cycle and had free access to standard laboratory chow and tap water.

Experiment 1: determine the best optimal timing of CD34^+^ cell treatment for the neuroprotection against focal cerebral ischemia

Forty eight ovariectomized female Sprague Dawley rats (270–320 g) were randomly assigned to 4 groups (n = 12 in each group): (1) sham operation, (2) MCAO with no CD34^+^ cell treatment (no treatment group), (3) infusion with 1×10^6^ CD34^+^ cells 30 min before MCAO (pre-treatment group) and (4) infusion with 1×10^6^ CD34^+^ cells 30 min after MCAO (post-treatment group). To induce ovariectomized status, all rats underwent bilateral salpingo-oophorectomy through a lower abdominal midline incision using a sterile technique 2 weeks before MCAO. The timing of CD34^+^ cell treatment which induced the least infarction volume in experiment 1 was selected for the experiment 2.

Experiment 2: evaluate the protective effect of CD34^+^ cells + estradiol treatment against focal cerebral ischemia

Sixty ovariectomized female Sprague Dawley rats (270–320 g) were randomly assigned to 5 groups (n = 12 in each groups): (1) sham operation, (2) MCAO with no treatment (no treatment group), (3) MCAO with infusion of 17β-estradiol (E_2_, 500 μg/kg) (E_2_ pre-treatment group), (4) MCAO with infusion of 1×10^6^ CD34^+^ cells (CD34^+^ pre-treatment group), and (5) MCAO with infusion of E_2_ (500 μg/kg) and 1×10^6^ CD34^+^ cells (E_2_+CD34^+^ pre-treatment group). Cerebral T2-weighted MR images (T2WI) were studied to characterize the extent of infarction volume at 2 h and 2 d after MCAO (n = 6 at each time point) in each group. After MR imaging study, rats were anesthetized with 3% isoflurane in a mixture of air with nitrous oxide and oxygen (70%/30%) before being euthanized. Rats were quickly decapitated and their brains were removed for the immunohistochemistry study of c-Fos, c-Jun, GFAP and BDNF.

### Induction of focal cerebral ischemia

Transient left MCA occlusion for 60 min was used as an acute ischemic model according to our previous method [17]. Before surgical procedure, rats were quickly anesthetized after inhalation of 3% isoflurane in a mixture of air with nitrous oxide and oxygen (70%/30%), and then 1.5% isoflurane was maintained during the experiment. The proximal portion of external carotid artery (ECA) was tightly ligated with a silk suture. A 20-mm 4–0 nylon surgical thread was inserted from left ECA into internal carotid artery (ICA) to occlude the MCA. Left common carotid artery (CCA) was then permanently ligated and the wound was temporarily closed. Anesthesia was discontinued after these procedures were completed. After a 60-min occlusion of left MCA, rat was anesthetized again, and the wound was opened to remove nylon surgical thread to recanalize MCA. In sham-operation group, similar procedures were conducted without ligation or occlusion of any vessel. Endovascular suture occlusion of MCA for 60 min could result in irreversible cerebral ischemic injury in both cerebral cortex and striatum [17]. Therefore, we only included animals that exhibited right-side weakness with upper-limb dominance and had infarction in both striatum and cortex at 2 d for study. In both experiments 1 and 2, a total of 127 rats were used. Among them, 3 rats were found to have intracranial hemorrhage after decapitation which was assumed due to penetration of MCA by nylon thread, 5 had no right-side weakness and cerebral infarction, 2 died within 2 days after operation, 2 died during operation, and 15 had only striatal infarction detected by T2-weighted image on the second day after MCAO (n = 6 in experiment 1 and n = 9 in experiment 2). Although a total of 27 rats were excluded, the final number for each group was kept at 6 for statistical analysis.

### Preparation of HUCB derived CD34^+^ cells

After the approval by Institutional Review Board of Chang Gung Memorial Hospital, HUCB was obtained from healthy adults with written informed consent using a VarioMACS Starting Kit (Miltenyi Biotec, Bergisteh Gladbach, Germany) and CD34 MicroBead Kit (Miltenyi Biotec, Bergisteh Gladbach, Germany) according to the manufacturer’s protocol. The CD34^+^ cells were collected and washed with phosphate-buffered saline (PBS), and then prepared to a final concentration of 1×10^6^ cells/0.3 mL in PBS [18]. Thirty minutes before and after MCAO, 1×10^6^ collected CD34^+^ cells were administered via femoral vein under inhalation anesthesia. The CD34^+^ purification was technically supported by Research and Development Department, HealthBanks Biotech Co., Ltd, and the quantity of CD34^+^ cells was calculated according to previous report [19].

### Magnetic resonance imaging

As described in detail previously [20], inhalation of a nitrous oxide/oxygen/isoflurane mixture (70%/30%/1.5%) was used to anesthetize the rats before MRI. After exposing the inguinal area, a biomedical silicone catheter (external diameter of 0.64 mm) was cannulated into left femoral vein to administer MR contrast agent. During MR scanning, rats were placed in prone position with head fixed in a reproducible position in a nonmagnetic cradle by using an appropriate spacer.

The MR images were obtained using a 7T Clinscan animal MRI system with a bore size of 30 cm (Bruker, Ettlingen, Germany). A volume resonator with a diameter of 72 mm was used for radio frequency transmission, and a 4-channel phased array coil optimized for rat brains was used to receive signals. A T2-weighted turbo spin echo sequence was applied to cover most of the brain regions of interest with 20 coronal slices that used the following imaging parameters: TR/TE = 2920 ms/38 ms, ETL = 7, slice thickness = 1 mm, matrix size = 256 × 256, in-plane resolution = 0.148 × 0.148 mm^2^. From these images, five slice locations (2-mm thickness) were selected based on a bregma of 0.4 mm and an interaural distance of 8.6 mm for T2-weighted imaging with set imaging parameters used for diffusion-tensor imaging (DTI) and dynamic susceptibility contrast (DSC) MRI. DTI used a spin-echo echo-planar imaging (EPI) sequence (TR/TE = 3000 ms/34 ms, matrix size = 128 × 128, in-plane resolution = 0.3 × 0.3 mm^2^, averages = 3) with diffusion gradients applied in 30 directions (b = 600 and 1200 mm/s^2^). Parallel imaging was applied using generalized auto-calibrating partially parallel acquisitions with an acceleration factor of 2. The apparent diffusion coefficient (ADC) maps were calculated using the DTI images. Perfusion weighted imaging was conducted using a gradient-echo EPI sequence (TR/TE/FA = 600 ms/10 ms/50°, matrix size = 128 × 128, in-plane resolution = 0.3 × 0.3 mm, acceleration factor = 2). An amount of 0.3 mL MR contrast media (0.15 mmol dimeglumine gadopentetate contrast; Magnevist, Bayer Schering Pharma, Berlin, Germany) was administered in 3 seconds at the seventh measurement. DSC-MRI data were processed using nICE software (Nordic ICE, NordicNeuroLab, Bergen, Norway). The arterial input function was semi-automatically obtained from voxels around right MCA (contralateral to the occlusion side) in each animal, based on criteria including peak height and rising time of concentration time curve. Relative CBF (relCBF) maps were then generated using a SVD deconvolution method [18,21]. Brain MR imagines were longitudinally examined in all rats 1 d before and 2 h and 2 d after MCAO.

### Immunohistochemistry

Animals were decapitated at 2 h and 2 d immediately after brain MR scanning. Brains were dissected out, frozen in powdered dry ice and stored at -80°C. Immunostaining against BDNF, c-Fos, c-Jun and GFAP in fresh-frozen brain sections was performed with avidin-biotin peroxidase method using a kit (PK-6102, Vector Labs, Burlingame, CA) [18,22,23]. Briefly, coronal sections (20 μm) at the level of striatum covering infarcted brain region of 12 mm in length were cut on a cryostat at -18°C, and the sections were mounted on glass microscope slides coated with saline (Muto Pure Chemical, Tokyo, Japan) and stored at -80°C until immunostaining. For immunohistochemistry, a total of 24 sections with an interval of 500 μm were used. After the fresh frozen sections were fixated in ice-cold acetone, they were incubated overnight at 4°C with the first antibodies and were then reacted for 1 h with the biotinylated second antibodies. We used 3,3'-diaminobenzidine tetrahydrochloride and 0.02% H_2_O_2_ for tissue staining. The average optical density of immunoreactivity at lesion and non-lesion striatum and cortex was calculated from the 24 sections per rat, and six rats at each time point were included for analysis. Negative control sections were prepared from the same tissue blocks by omitting the specific primary antibodies and using normal, non-immune serum supernatant from the same sources.

Image-Pro Plus Software (Media Cybernetics, Silver Spring, MD) was used for immunoreactivity calculations. The ratio of the optical density of no treatment and pre-treatment MCAO rat to the mean value of sham-operation rats was determined for BDNF, c-Fos, c-Jun and GFAP analyses.

### Statistical Analysis

The data were analyzed statistically using one-way analysis of variance test followed by Tukey test. All data used Prism 5 software for statistical analysis (GraphPad, San Diego, CA). Values were considered significant at *p <* 0.05.

## Results

### Experiment 1

**Fig 1:**
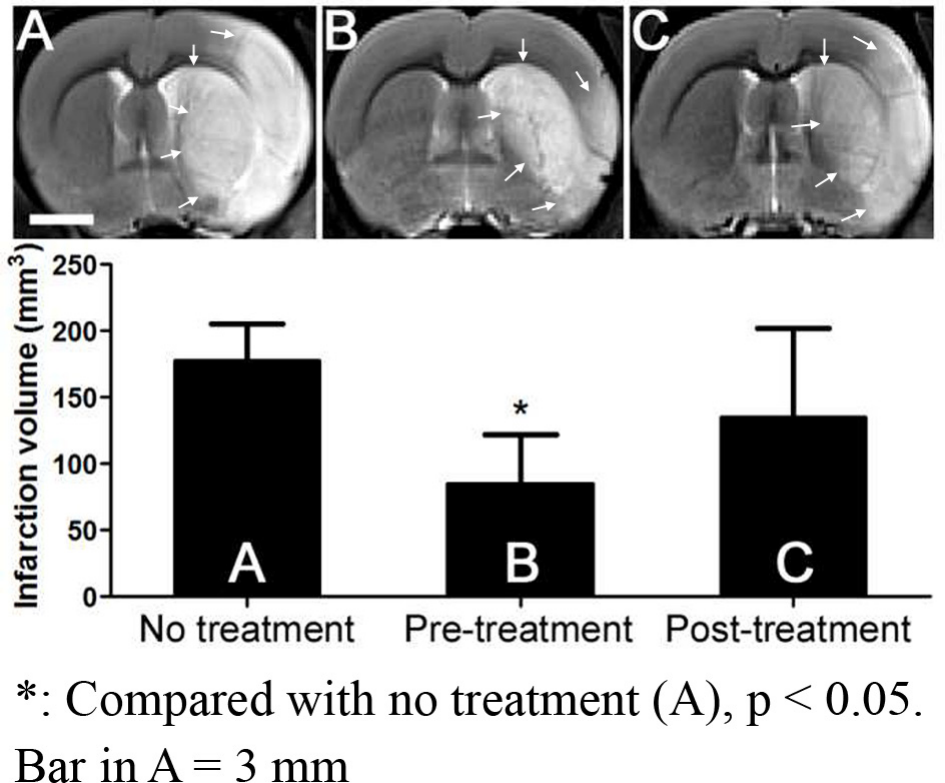
Representative T2-weighted images obtained 2 days after middle cerebral artery occlusion in the 3 groups of rat treated with CD34^+^. Infarction volume is significantly smaller in pre-treatment groups than no treatment group, and no significant difference of infarction volume between pre-treatment and post-treatment groups and between post-treatment and no treatment groups. Arrows indicate the margin of infarction area.

The result showed the infarction volume was significantly smaller in CD34^+^ pre-treatment group (84.94 ± 37.14 mm^3^) than no treatment group (177.22 ± 28.39 mm^3^, p = 0.011) on T2-weighted images obtained at 2 d after MCAO (Fig 1). There was no significant difference of infarction volume between CD34^+^ pre-treatment and CD34^+^ post-treatment groups (134.82 ± 66.59 mm^3^) and between post-treatment and no treatment groups (p > 0.05).

### Experiment 2

**Fig 2:**
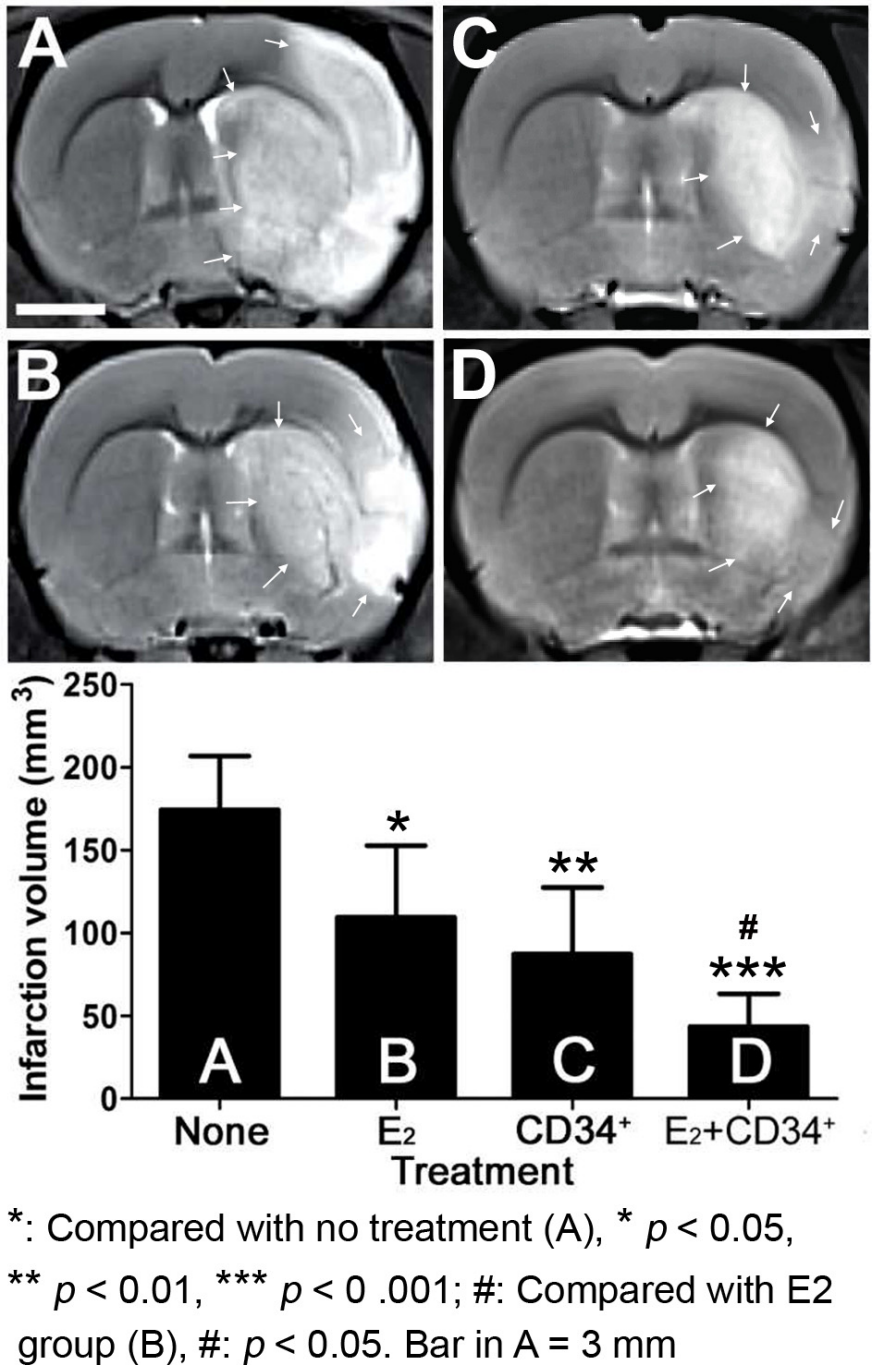
Representative T2-weighted images obtained 2 days after middle cerebral artery occlusion in no treatment and the 3 pre-treatment groups. Infarction volume is significantly reduced in the 3 pre-treatment groups when compared with no treatment group. Arrows indicate the margin of infarction area. Among the three pre-treatment groups, pre-treatment with E_2_+CD34^+^ has the least infarction volume and shows significant difference when compared with E_2_ pre-treatment groups.

The result showed the infarction volume examined on T2-weighted images at 2 d after MCAO was significantly reduced in the 3 pre-treatment groups (E_2_: 109.54 ± 43.29 mm^3^, CD34^+^: 87.14 ± 40.20 mm^3^, E_2_+CD34^+^: 43.34 ± 20.04 mm^3^) when compared with no treatment group (174.15 ± 32.55 mm^3^, p = 0.026, 0.003, and < 0.001, respectively) (Fig 2). Among the three pre-treatment groups, pre-treatment with E_2_+CD34^+^ had the least infarction volume and showed significant difference when compared with E_2_ pre-treatment groups (p = 0.031).

### Apparent diffusion coefficient (ADC)

**Fig 3:**
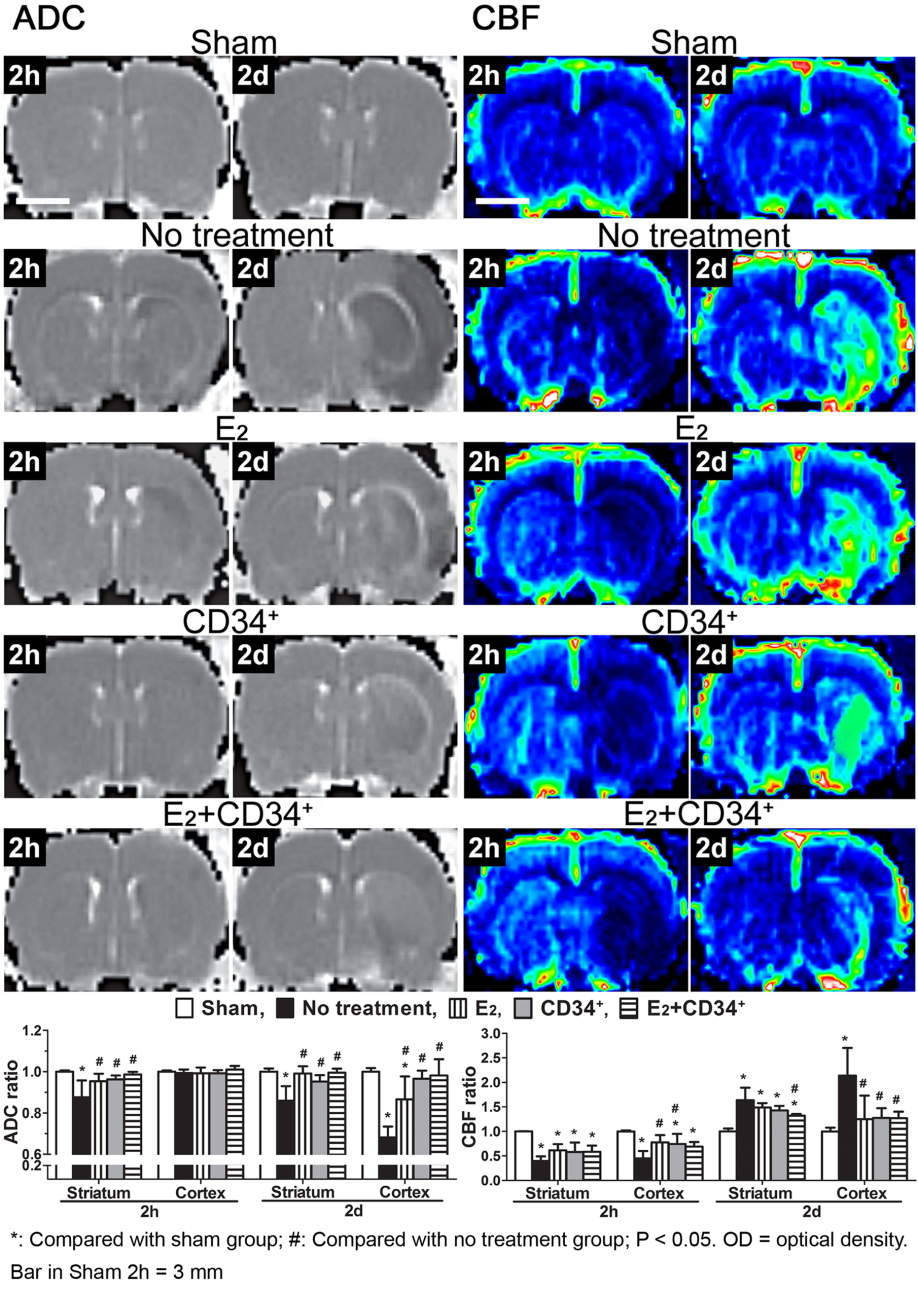
Temporal changes of apparent diffusion coefficient (ADC) and cerebral blood flow (CBF) after middle cerebral artery occlusion in no treatment and the 3 pre-treatment groups. Fig 3 ADC: When compared with sham-operation group, ADC shows significant reduction from 2 h to 2 d in striatum and at 2 d in cortex of no treatment and E_2_ groups. However, when compared with no treatment group, ADC shows less reduction from 2 h to 2 d in striatum and at 2 d in cortex of the 3 pre-treatment groups. Fig 3 CBF: When compared with sham-operation group, CBF demonstrates significant reduction at 2 h in both striatum and cortex (except E_2_ group) and significant increase at 2 d in striatum of no treatment and the 3 pre-treatment groups but only in cortex of no treatment group. However, when compared with no treatment group, CBF demonstrates significant recovery at 2 h in cortex after E_2_ or CD34^+^ pre-treatment but has less hyperperfusion above sham-operation level at 2 d in striatum of E_2_+CD34^+^ group and in cortex of the 3 pre-treatment groups.

When compared with sham-operation group, ADC showed significant reduction from 2 h to 2 d in striatum (both p < 0.001) and at 2 d in cortex of no treatment and E_2_ groups (p < 0.001 and p = 0.017, respectively). However, when compared with no treatment group, ADC showed less reduction from 2 h to 2 d in striatum (p < 0.05) and at 2 d in cortex of the 3 pre-treatment groups (p < 0.05) (Fig 3 ADC).

### Cerebral blood flow (CBF)

When compared with sham-operation group, CBF demonstrated significant reduction at 2 h in both striatum and cortex (except E_2_ group) and significant increase at 2 d in striatum of no treatment and the 3 pre-treatment groups (p < 0.05) but only in cortex of no treatment group (p = 0.004). However, when compared with no treatment group, CBF demonstrated significant recovery at 2 h in cortex after E_2_ or CD34^+^ pre-treatment (p = 0.013 and 0.031, respectively) and had less hyperperfusion above sham-operation level at 2 d in striatum of E_2_+CD34^+^ group (p = 0.013) and in cortex of the 3 pre-treatment groups (p < 0.05) (Fig 3 CBF).

### c-Fos and c-Jun

**Fig 4:**
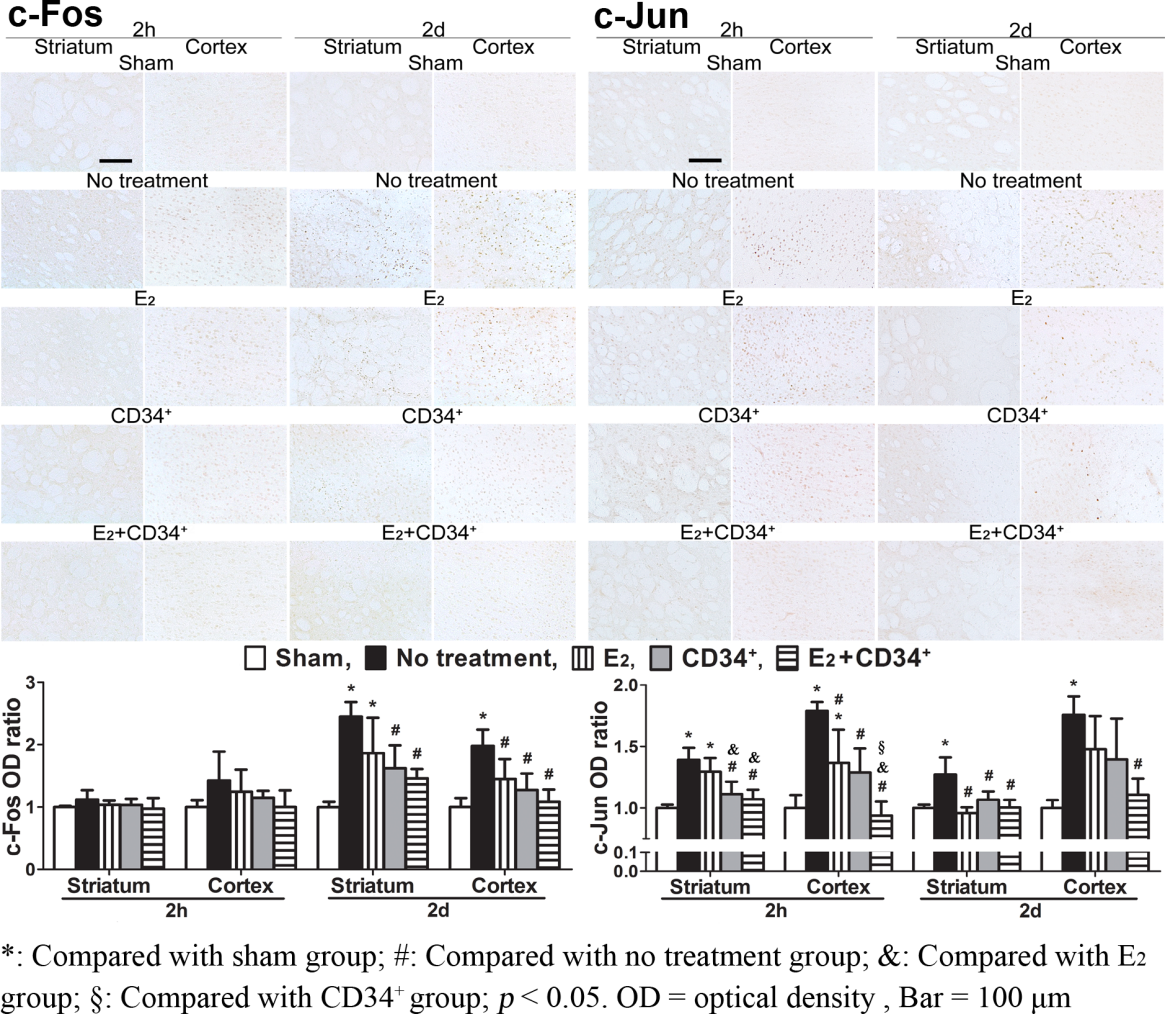
Temporal expressions of c-Fos and c-Jun immunoreactivity after middle cerebral artery occlusion in no treatment and the 3 pre-treatment groups. When compared with sham-operation group, c-Fos is significantly expressed at 2 d in striatum of no treatment and E_2_ groups and in cortex of no treatment group. However, when compared with no treatment group, c-Fos is less expressed at 2 d in striatum of CD34^+^ and E_2_+CD34^+^ groups and in cortex of the 3 pre-treatment groups. When compared with sham-operation group, c-Jun is significantly expressed from 2 h in both striatum and cortex of no treatment and E_2_ groups to 2 d in both striatum and cortex of no treatment group. However, when compared with no treatment group, c-Jun is less expressed from 2 h in striatum of CD34^+^ and E_2_+CD34^+^ groups to 2 d in striatum of the 3 pre-treatment groups and from 2 h in cortex of the 3 pre-treatment groups to 2 d in cortex of E_2_+CD 34^+^ group. Among the three pre-treatment groups, pre-treatment with E_2_+CD34^+^ has the least expression of c-Fos and c-Jun and shows significant reduction of c-Jun when compared with CD34^+^ and/or E_2_ pre-treatment groups at 2 h.

When compared with sham-operation group, c-Fos was significantly expressed at 2 d in striatum of no treatment and E_2_ groups and in cortex of no treatment group (all p < 0.01). However, when compared with no treatment group, c-Fos was less expressed at 2 d in striatum of CD34^+^ and E_2_+CD34^+^ groups and in cortex of the 3 pre-treatment groups (all p < 0.05). When compared with sham-operation group, c-Jun was significantly expressed from 2 h in both striatum and cortex of no treatment and E_2_ groups to 2 d in both striatum and cortex of no treatment group (all p < 0.05). However, when compared with no treatment group, c-Jun was less expressed from 2 h in striatum of CD34^+^ and E_2_+CD34^+^ groups to 2 d in striatum of the 3 pre-treatment groups and from 2 h in cortex of the 3 pre-treatment groups to 2 d in cortex of E_2_+CD 34^+^ group (all p < 0.05). Among the three pre-treatment groups, pre-treatment with E_2_+CD34^+^ had the least expression of c-Fos and c-Jun and showed significant reduction of c-Jun when compared with CD34^+^ and/or E_2_ pre-treatment groups at 2 h (striatum: E_2_ vs. E_2_+CD34^+^, E_2_ vs. E_2_+CD34^+^, and CD34^+^ vs. E_2_+CD34^+^: all p < 0.05) (Fig 4).

### Glial fibrillary acid protein (GFAP)

**Fig 5:**
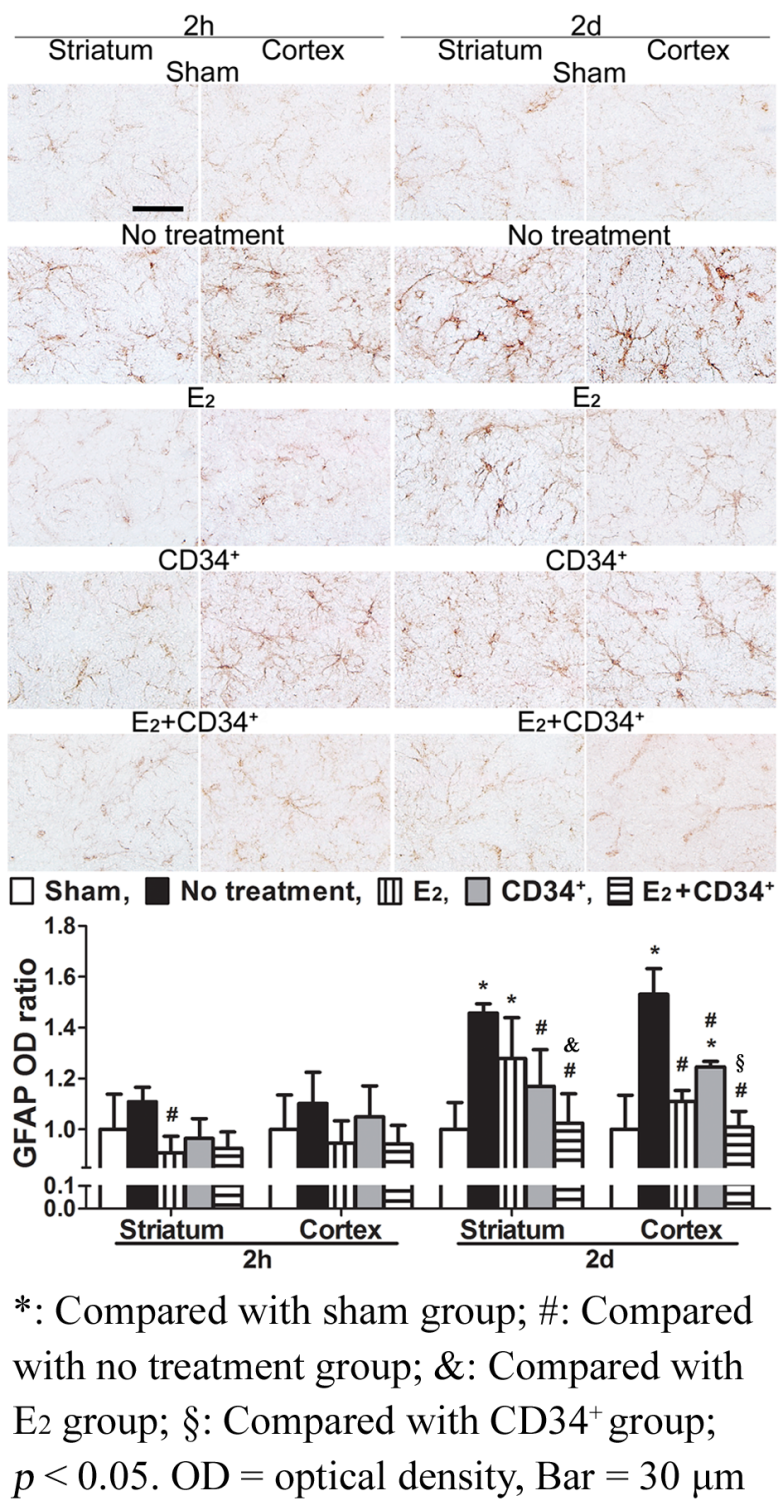
Temporal expression of glial fibrillary acid protein (GFAP) immunoreactivity after middle cerebral artery occlusion in no treatment and the 3 pre-treatment groups. When compared with sham-operation group, GFAP is significantly expressed at 2 d in striatum of no treatment and E_2_ groups and in cortex of no treatment and CD34^+^ groups. When compared with no treatment group, GFAP is less expressed at 2 h in striatum of E_2_ group and less expressed at 2 d in striatum of CD34^+^ and E_2_+CD34^+^ groups and in cortex of the 3 pre-treatment groups. Among the three pre-treatment groups, pre-treatment with E_2_+CD34^+^ has the least expression of GFAP and shows significant reduction when compared with E_2_ in striatum and CD34^+^ in cortex at 2 d.

When compared with sham-operation group, GFAP was significantly expressed at 2 d in striatum of no treatment and E_2_ groups and in cortex of no treatment and CD34^+^ groups (all p < 0.05). When compared with no treatment group, GFAP was less expressed at 2 h in striatum of E_2_ group and less expressed at 2 d in striatum of CD34^+^ and E_2_+CD34^+^ groups and in cortex of the 3 pre-treatment groups (all p < 0.05). Among the three pre-treatment groups, pre-treatment with E_2_+CD34^+^ had the least expression of GFAP and showed significant reduction when compared with E_2_ in striatum and CD34^+^ in cortex at 2 d (all p < 0.05) (Fig 5).

### Brain-derived neurotrophic factor (BDNF)

**Fig 6:**
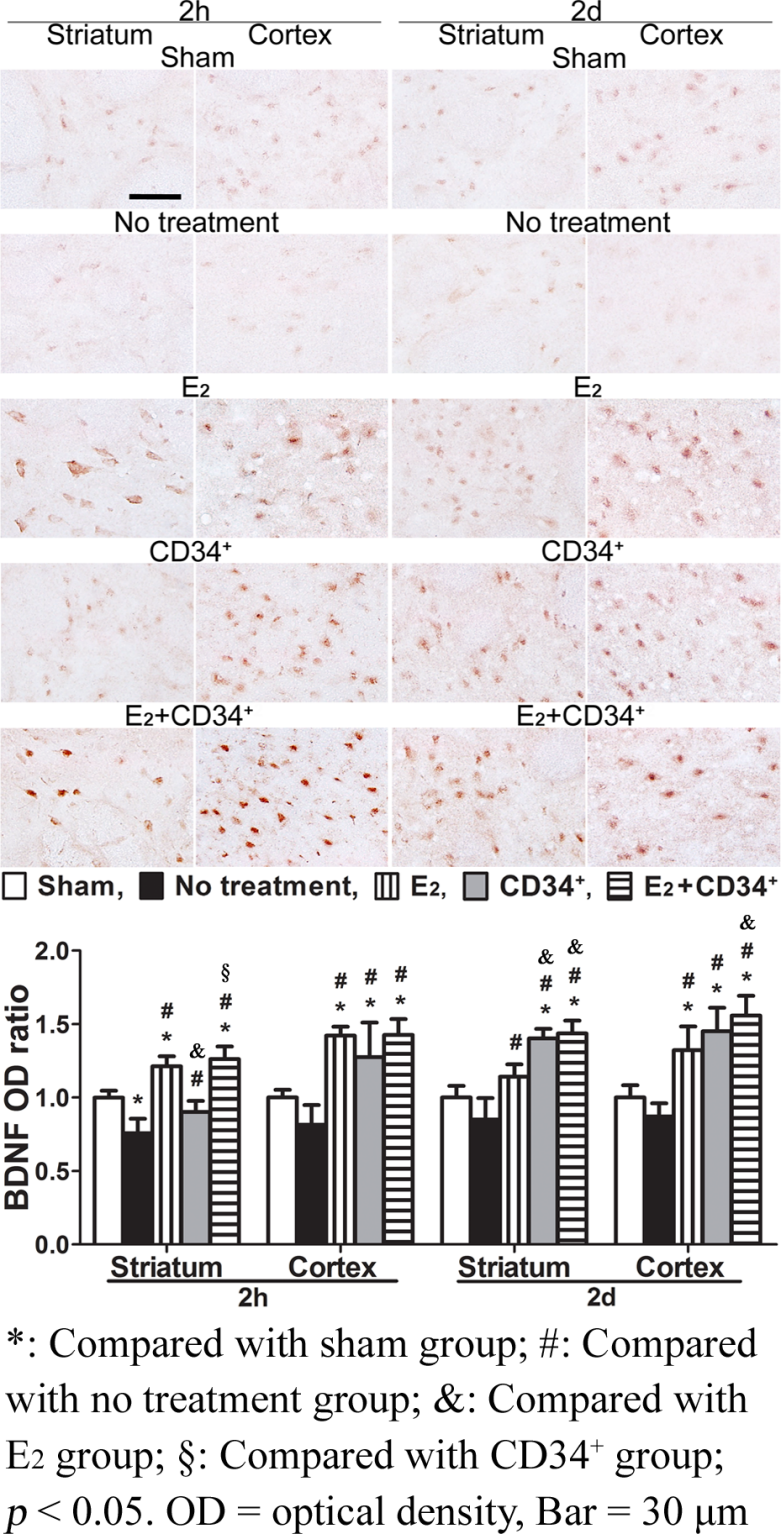
Temporal expression of brain-derived neurotrophic factor (BDNF) immunoreactivity after middle cerebral artery occlusion in no treatment and the 3 pre-treatment groups. When compared with sham-operation group, BDNF immunoreactivity is significantly reduced at 2 h in striatum of no treatment group but is significantly increased at 2 h in striatum of E_2_ and E_2_+CD34^+^ groups. There is also significant increase at 2 d in striatum of CD34^+^ and E_2_+CD34^+^ groups and from 2 h to 2 d in cortex of the 3 pre-treatment groups. When compared with no treatment group, BDNF immunoreactivity shows significant recovery from 2 h to 2 d in both striatum and cortex of the 3 pre-treatment groups. Among the three pre-treatment groups, pre-treatment with E_2_+CD34^+^ has the highest expression of BDNF and shows significant increase when compared with CD34^+^ in striatum at 2 h and E_2_ in striatum and cortex at 2 d.

When compared with sham-operation group, BDNF immunoreactivity was significantly reduced at 2 h in striatum of no treatment group but significantly increased at 2 h in striatum of E_2_ and E_2_+CD34^+^ groups (all p < 0.001). There was also significant increase at 2 d in striatum of CD34^+^ and E_2_+CD34^+^ groups (both p < 0.001) and from 2 h to 2 d in cortex of the 3 pre-treatment groups (p < 0.05). When compared with no treatment group, BDNF immunoreactivity showed significant recovery from 2 h to 2 d in both striatum and cortex of the 3 pre-treatment groups (p < 0.05). Among the three pre-treatment groups, pre-treatment with E_2_+CD34^+^ had the highest expression of BDNF and showed significant increase when compared with CD34^+^ in striatum at 2 h (p < 0.001) and with E_2_ in striatum (p = 0.001) and cortex (p = 0.041) at 2 d (Fig 6).

## Discussion

Recent studies found that HUCB-derived CD 34^+^ cells might be an effective fraction of cord blood cells to promote functional recovery through angiogenesis and neurogenesis [2,3]. Taguchi et al. [2,3] reported that systemic administration of CD34^+^ cells to MCAO mice could induce neovascularization in ischemic zone followed by endogenous neurogenesis, suggesting the potential role of neovascularization for neuronal regeneration and functional recovery after stroke. Nevertheless, Nystedt et al. [2] reported intravenous administration of CD34^+^ cells after MCAO might improve functional outcome in rats but did not significantly provide neuroprotection, because CD 34^+^ cells did not reduce infarction size in either transient or permanent MCAO. Our study revealed pre-treatment of CD34^+^ cells can be more effective than post-treatment to reduce infarction volume after MCAO, which is further enhanced with complemental estrogen treatment in ovariectomized rat.

Estrogen replacement therapy in postmenopausal women may improve cognitive function and reduce neurodegeneration in Alzheimer's disease and risk of stroke. Animal study showed estrogens may reduce mortality and ischemic damage caused by MCAO in ovariectomized rat [24] and decrease ischemia–induced lesion volume significantly when administered within 30 min after permanent MCAO [10,11]. Dubal et al. also reported significant reduction of overall infarction volume after permanent MCAO in ovariectomized rats with pre-treatment of estradiol [25]. Our results demonstrated that pre-treatment of estradiol has neuroprotective effect against ischemic injury, and co-treatment with estradiol and CD34^+^ cells can exert a synergic neuroprotective effect after MCAO in ovariectomized rats.

In the present study, we used MR diffusion and perfusion to study the effect of angiogenesis and neurogenesis of pre-treatment with CD34^+^ cell and/or estradiol. We found that compared with no treatment group, pre-treatment with CD34^+^ cell and/or estradiol could cause significant reduction of infarction volume (T2 image in Fig 2) and brain edema (ADC in Fig 3) after ischemic injury especially when CD34^+^ cell and estradiol were co-administrated. The recovery of the T2 signal in pre-treatment group may indicate a better resolution of vasogenic edema [16,26], which is typically caused by vascular regulatory dysfunction after blood-brain barrier disruption. The less ADC signal intensity in pre-treatment group than no treatment group also indicates resolution of cytotoxic edema [26,27], which is typically caused by cellular metabolic impairment after ischemia.

Delayed increase in MR signal intensity of both CBF and cerebral blood volume has been reported to occur from day 1 to 14 in the ipsilateral cortex after transient MCAO in rats [16]. Previous study revealed that postischemic hyperperfusion was neither correlated to the final infarction nor did it affect the outcome [28], whereas another study reported that postischemic hyperperfusion was associated with an increased infarction volume [27]. Similar findings have also been reported and were attributed to increased angiogenesis [16] and vascular regulatory dysfunction after ischemia [29]. Yang et al. [11] found estradiol exerts neuroprotective effect when administered after transient MCAO in ovariectomized rats, and this effect of estradiol was associated with no immediate change in blood flow but with a delayed increase in CBF. Our study revealed that when compared with no treatment group, there was less reduction of CBF at 2 h and less hyperperfusion above sham-operation level at 2 d in the 3 pre-treatment groups, suggesting a reduced postischemic hyperperfusion injury especially in E_2_+CD34^+^ pre-treatment group.

Previous report showed selected proto-oncogenes including c-Fos and c-Jun could be increased after MCAO, and pre-treatment of estradiol was reported to differentially regulate c-Fos after ischemia in ovariectomized rats [14]. However, our study showed that pre-treatment of estradiol can not only attenuate the expression of c-Fos and c-Jun but also result in further attenuation when co-treated with CD34^+^ cells. The increase in c-Fos and c-Jun is found to regulate activator protein-1 binding activity in ischemic cortex, which in turn may link to the enhanced expression of neurotrophin genes [30]. Postischemic intracerebroventricular infusion of BDNF was reported to prevent neuronal death in vulnerable hippocampal CA1 region and inhibit astroglial activation and macrophage infiltration which were observed in association with neuronal death [7]. Our previous study revealed that the recovery of neurotrophin level in ischemic neurons is important for neuronal survival [31]. Our present result showed that BDNF immunoreactivity recovered well with significant attenuation of immediate early genes and reactive astrocytosis after ischemia in the 3 pre-treatment groups when compared with no treatment group.

Our study has some limitations. First, we did not examine the effect of different doses of CD34^+^ and estradiol and their combination. It is possible that higher dose of CD34^+^ or estradiol could be effective alone without the need of combination treatment. The combination of higher dose of CD34^+^ and estradiol could also be more effective than the combination of present dose. Second, we did not examine if post-treatment with higher dose of CD34^+^, estradiol or combination therapy at different time points could be also effective, since pre-treatment usually has limited therapeutic application. Third, we did not examine different pre- and post-treatment time points to study when could be the best moment to cause significant effectiveness. In the future study, these issues should be carefully examined.

## Conclusion

Our results demonstrate that the neuroprotective effect of intravenous administration of HUCB derived CD34^+^ cells may act through stabilization of cerebral hemodynamics and normalization of the expression of immediate early genes and BDNF after focal cerebral ischemia. Estradiol can add a synergic effect to CD34^+^ cells in the mechanism of neuroprotection.
